# COVID-19 vaccination in prison settings: a model to design tailored vaccine delivery strategies

**DOI:** 10.1093/eurpub/ckac129.388

**Published:** 2022-10-25

**Authors:** L Tavoschi, S Mazzilli, D Petri, V Busmachiu, I Stylianou, F Meroueh, H Stöver, A Rosello, R Ranieri, L Baglietto

**Affiliations:** 1 Department of Translational Research in Medicine, University of Pisa, Pisa, Italy; 2 Scuola Normale Superiore, Pisa, Italy; 3 Department of Clinical and Experimental Medicine, University of Pisa, Pisa, Italy; 4 National Administration of Penitentiaries, Chișinău, Moldova; 5 Cyprus Prison Department, Ministry of Justice and Public Order, Nicosia, Cyprus; 6 Centre Hospitalier Universitaire Montpellier, Montpellier, France; 7 Institute of Addiction Research, University of Applied Sciences, Frankfurt am Main, Germany; 8 UK Health Security Agency, London, UK; 9 ASST Santi Paolo e Carlo, San Paolo Hospital, Milan, Italy

## Abstract

**Introduction:**

Vaccinations are one of the most powerful preventive tools discovered by modern medicine. Although expanded programmes of immunization are well established in EU/EEA, significant immunity gaps and suboptimal coverage are registered among specific populations, including people living in prisons (PLP). PLP are also at increased risk to vaccine-preventable diseases (VPD) with potential outbreak in prison, e.g. flu, COVID-19, as well as other VPDs such as HBV. The EU-funded project RISE-Vac, aimed at collecting models of care developed during the pandemic to design tailored vaccine delivery strategies that could be extended beyond the sole COVID-19 vaccine.

**Methods:**

Through a survey to healthcare staff working in prisons in six countries of the EU/EEA (Cyprus, France, Germany, Italy, Moldova, UK) we collected information on the implementation of COVID-19 vaccination program. The following areas were investigated: challenges & barriers encountered, workload distribution, education & training activities for prison staff and PLP, referral strategies after release, immunization information system.

**Results:**

The respondents reported that in prisons COVID-19 programs have been implemented efficiently. Strategies for optimal management of the vaccination campaign included: week-day dedicated to vaccination services when vaccines were delivered and immediately administered to overcome cold chain challenges; new staff recruitment & task shifting; administration of booster doses within prison premises for released individuals; distribution of informational material both to PLP & prison staff.

**Conclusions:**

Our results show that universal immunisation campaigns are feasible, acceptable and effective in places of detention when there is commitment to implementing them. Evidence from the pandemic situation may inform future provision of expanded immunization programmes.

